# The Calcium Goes Meow: Effects of Ions and Glycosylation on Fel d 1, the Major Cat Allergen

**DOI:** 10.1371/journal.pone.0132311

**Published:** 2015-07-02

**Authors:** Rodrigo Ligabue-Braun, Liana Guimarães Sachett, Laércio Pol-Fachin, Hugo Verli

**Affiliations:** 1 Centro de Biotecnologia, Universidade Federal do Rio Grande do Sul (UFRGS), Porto Alegre, RS, Brazil; 2 Departamento de Química Fundamental, Universidade Federal de Pernambuco, Recife, PE, Brazil; Weizmann Institute of Science, ISRAEL

## Abstract

The major cat allergen, Fel d 1, is a structurally complex protein with two N-glycosylation sites that may be filled by different glycoforms. In addition, the protein contains three putative Ca^2+^ binding sites. Since the impact of these Fel d 1 structure modifications on the protein dynamics, physiology and pathology are not well established, the present work employed computational biology techniques to tackle these issues. While conformational effects brought upon by glycosylation were identified, potentially involved in cavity volume regulation, our results indicate that only the central Ca^2+ ^ion remains coordinated to Fel d 1 in biological solutions, impairing its proposed role in modulating phospholipase A_2_ activity. As these results increase our understanding of Fel d 1 structural biology, they may offer new support for understanding its physiological role and impact into cat-promoted allergy.

## Introduction

Allergic diseases, especially those involving responses mediated by immunoglobulin E (IgE), are increasing in prevalence and becoming major public health issues [1–3]. This increase has been associated with a Westernized life style and urbanization, suggesting that pets could contribute to this scenario as sources of indoor allergens [1, 4]. The most implicated pets in allergic diseases are cats (*Felis domesticus*), which are present in up to 50% of homes [5]. The sensitization prevalence in adults is in the range of 10–15%, with symptoms varying from rhinoconjunctivitis to potentially life-threatening asthmatic exacerbations [6].

**Fig 1:**
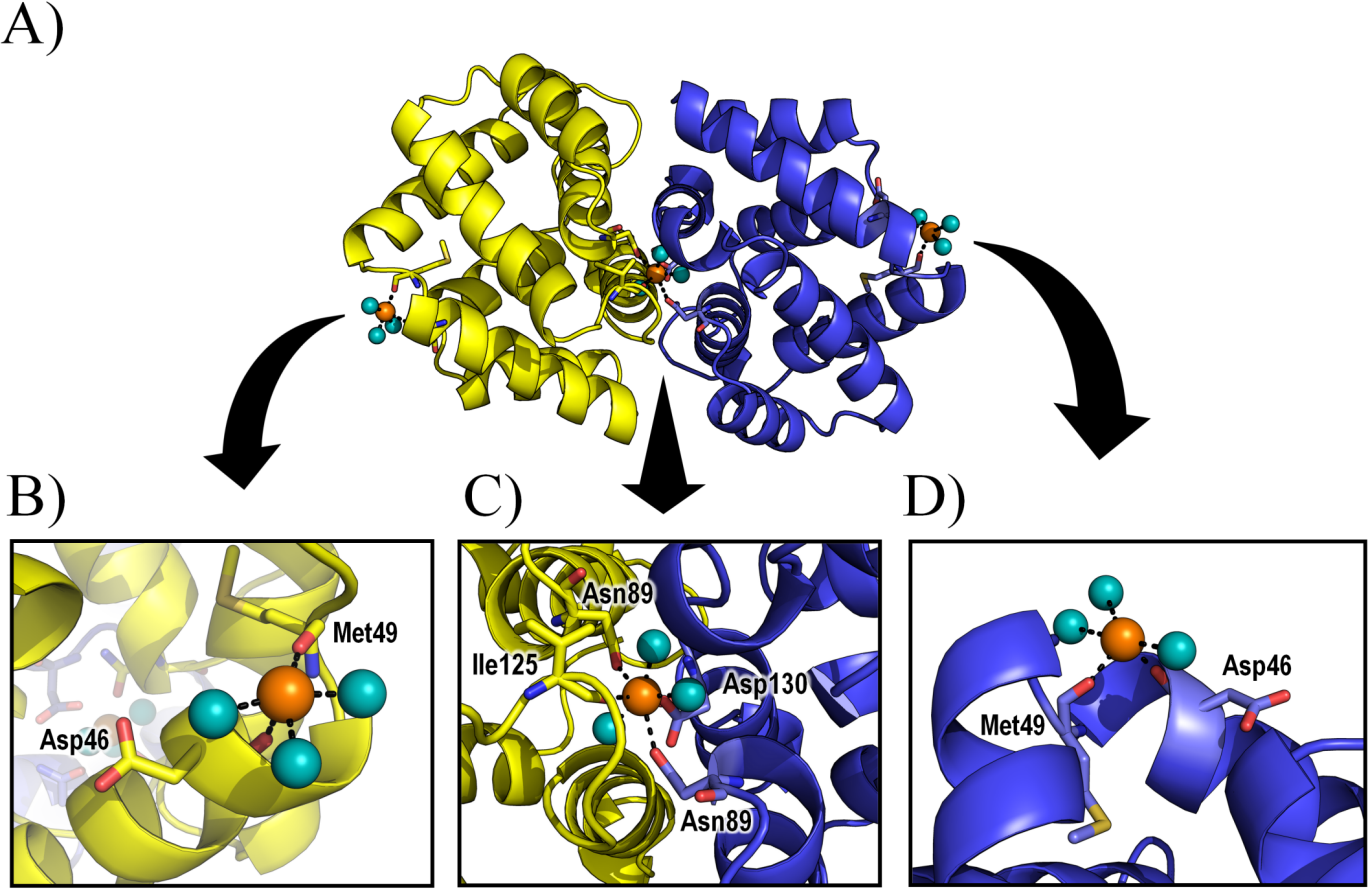
Fel d 1 Ca^2+^ binding sites. (A) Fel d 1 crystallographic structure highlighting the location of calcium ions; (B) Lateral Ca^**2+**^ binding site A; (C) Interfacial Ca^**2+**^ binding site; (D) Lateral Ca^**2+**^ binding site B. Proposed coordination interactions based on the crystallographic structure are shown as dashed lines. Calcium ions are shown as orange spheres, water molecules are shown as cyan spheres, Chain A is shown in yellow, Chain B is shown in blue. (the orientation of the boxes is different from the one in (A) for clarity).

Among the few components of cat dander that can elicit IgE response, the Fel d 1 protein is considered the most potent [6]. Fel d 1, the major cat allergen, is a dimer of all-helical heterodimers, included in the secretoglobin family [7, 8]. Each dimer in the native heterotetramer is N-glycosylated [9], and crystallographic analyses of recombinant Fel d 1 revealed that each dimer has a cavity, possibly involved in the transport of an unknown molecule [7, 8]. For crystallization, a fused version of the protein was produced, in which chains 1 and 2 are linked, making the original tetramer (or dimer of dimers), a simple dimer of the fused protein [8] (Fig 1).

The crystallographic study of this recombinant Fel d 1 tetramer also revealed three putative calcium-binding sites, one at the tetramerization interface and one on the lateral of each dimer [8]. The lateral binding sites involve O atoms from Asp46 and Met49 (main chain) and three water molecules within coordination distance from the Ca^2+^, while the binding site at the interface comprises OD1 atoms from Asn89 (from both chains A and B) and Asp130 (chain B), a carbonyl from Ile125 (chain A) and three water molecules [8] (Fig 1). The non-interfacial binding sites are proposed to act on allergic responses by modulating phospholipase A_2_ activity via calcium sequestration, a property related to uteroglobins [8, 10]. The interaction of Fel d 1 and calcium is however subject of debate, since for uteroglobin (a member of the secretogolobin protein family) some authors identified calcium binding, while others identified the opposite [10–13].

The importance of Fel d 1 glycosylation in protein structure and immune response has also been somewhat controversial. While some studies show that different glycosylation patterns do not affect IgE production *in vitro* [9, 14, 15], a most recent study demonstrated that the mannose receptor has an essential role in internalizing Fel d 1. Mannose receptor cysteine rich domain recognizes the carbohydrates in Fel d 1 and *in vivo* assays showed that knockout mice for mannose receptor produced lower levels of immunoglobulins E and G [16].

**Fig 2:**
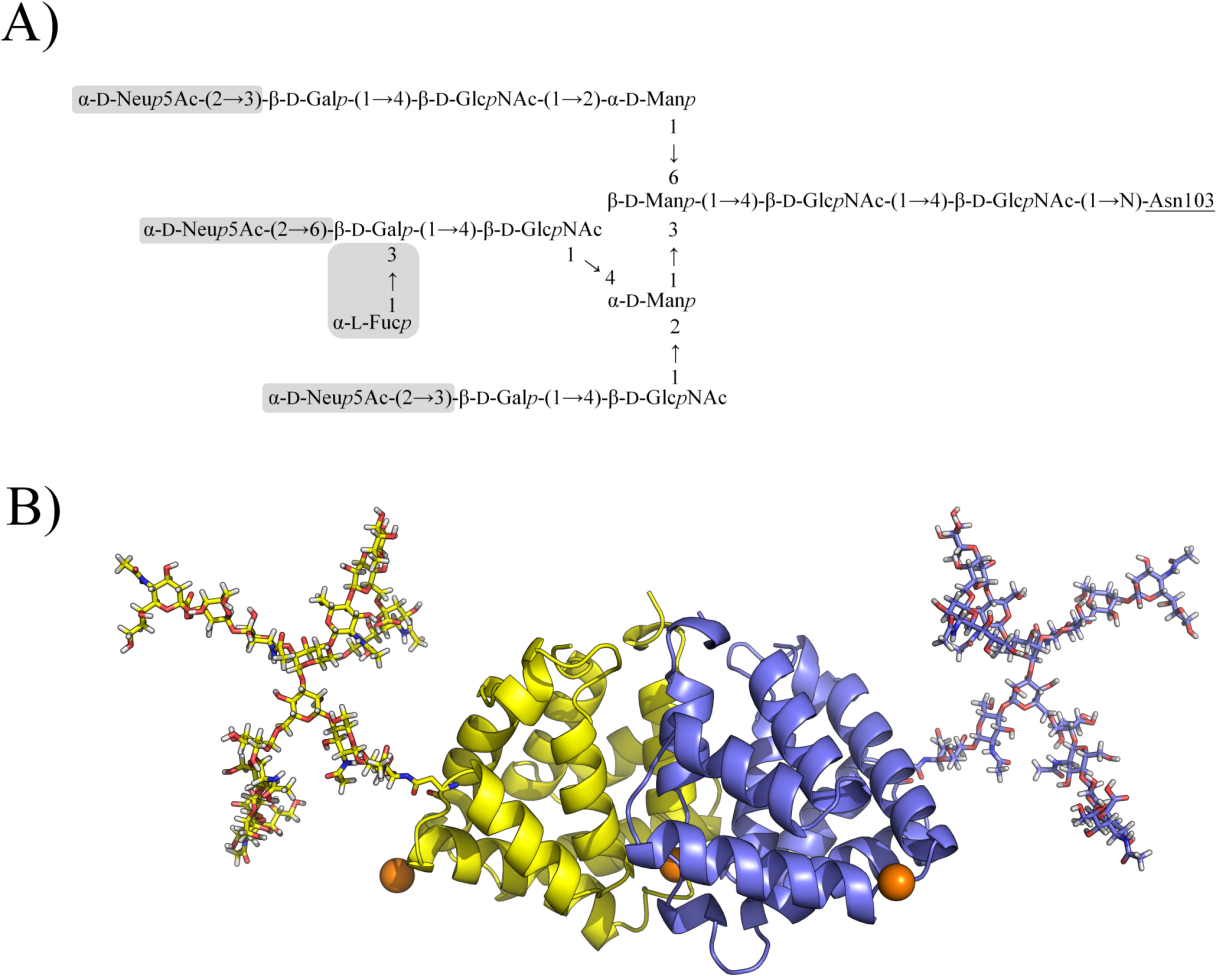
Fel d 1 glycosylation structures. (A) Full (largest) glycosylation structure of Fel d 1, shaded boxes indicate saccharides that are absent in the minimal (smallest) glycosylation structure. (B) Fel d 1 structure with full glycan chains attached. Calcium ions are shown as orange spheres, Chain A is shown in yellow, Chain B is shown in blue.

The glycosylation pattern of Fel d 1 has been determined by mass spectrometry, revealing a series of possible glycoforms bound to the protein [14]. As observed for other glycosylated proteins, there is a minimal structure to which different oligosaccharides may be added, leading to variation in the glycan moiety, which is limited to the largest glycosylation tree identified. For the present work we employed the extremes of the glycosylation structure, analyzing the largest (or “full glycosylation”) and the smallest (“minimal glycosylation”) structures found by mass spectrometry [14] (Fig 2). Intermediate glycostructures have different numbers of galactose, mannose, and sialic acid residues.

Despite the extensive studies on Fel d 1-induced allergy in humans (as reviewed by [6, 17]), very little information is available regarding its interactions with calcium ions or the effect of glycosylation. Considering that these properties may be important not only for allergy studies but also for understanding the physiological role of this protein in felines, the present work employed computational biology techniques to tackle these issues. Effects brought upon by glycosylation were identified, while the lateral calcium binding sites proposed by crystallography were not confirmed.

## Results

### Outline

In this work we performed molecular dynamics (MD) simulations of Fel d 1 using different force fields to evaluate a putative role of parameter differences in describing Ca^2+^ behavior in the protein environment. These molecular mechanics studies were complemented with semi-empirical calculations of the Ca^2+^ interaction with Fel d 1, providing electronic information regarding these ions. In a separate analysis, we inspected the role of two different glycosylation structures on the protein structure and dynamics. These glycoforms are the largest (full glycosylation) and smallest (minimal glycosylation) found on Fel d 1. Protein cavities and ligand-binding possibilities were also studied. The simulated systems and calculations are schematically listed below. Please refer to the Material and Methods section at the end of the article for version information, simulation details, and further references.

By MD we simulated the Fel d 1 dimer of fused dimers under three force fields (AMBER, CHARMM, GROMOS) for 200 ns, in order to guarantee that the Ca^2+^ orientation within Fel d 1 was not due to an specific set of parameters, as well as a simplified structural mockup of the crystallographic environment of Fel d 1 for 25 ns (GROMOS force field) in order to trace potential crystallographic contacts able to lock the Ca^2+^ in position. The role of glycan chains in Fel d 1 dynamics was evaluated through 200 ns simulations using GROMOS force field for two glycosylated systems (Fel d 1 in the largest and smallest glycoforms). After evaluation on the putative Ca^2+^ position dependence on force field parameters, the simulation of the glycoproteins employed GROMOS due to its robust set of parameters for glycoprotein simulations [18, 19], as well as to the lower computational cost associated to united atom force fields in comparison to all atom parameters. All MD simulations were carried out with the GROMACS simulation package. To include direct information on the ions degree of coordination, semi-empirical calculations of the Ca^2+^ binding sites were performed with MOPAC under three scenarios: with implicit solvent, without implicit solvent, and with the inclusion of crystallographic waters.

### Implementation and validation of GROMOS 53A6glyc sialic acid parameter

In order to enable Fel d 1 glycoforms to be studied by MD simulations, and considering that parameters for neuraminic (sialic) acid residues were absent in the original GROMOS 53A6glyc implementation, we have constructed a topology for such monosaccharide in the current work (S1 Table). It was built based on ring and hydroxyl parameters for aldohexopyranoses [18] under GROMOS 53A6glyc, as well as N-acetyl and carboxilate parameters previously employed in GROMOS 45A4/53A6glyc force fields [19, 20]. Before employing such parameters for fully glycosylated Fel d 1 MD simulations, we have validated such topology by evaluating isolated sialic acid conformational ring pucker properties, as performed for other monosaccharides [18]. Thus, while metadynamics calculations indicated ^2^ *C*_5_ as the preferential ring pucker conformation for sialic acid, unbiased 1000 ns MD simulations confirmed such behavior, in accordance with previous observations (S1 Fig).

### Analyzes of putative calcium binding sites

**Fig 3:**
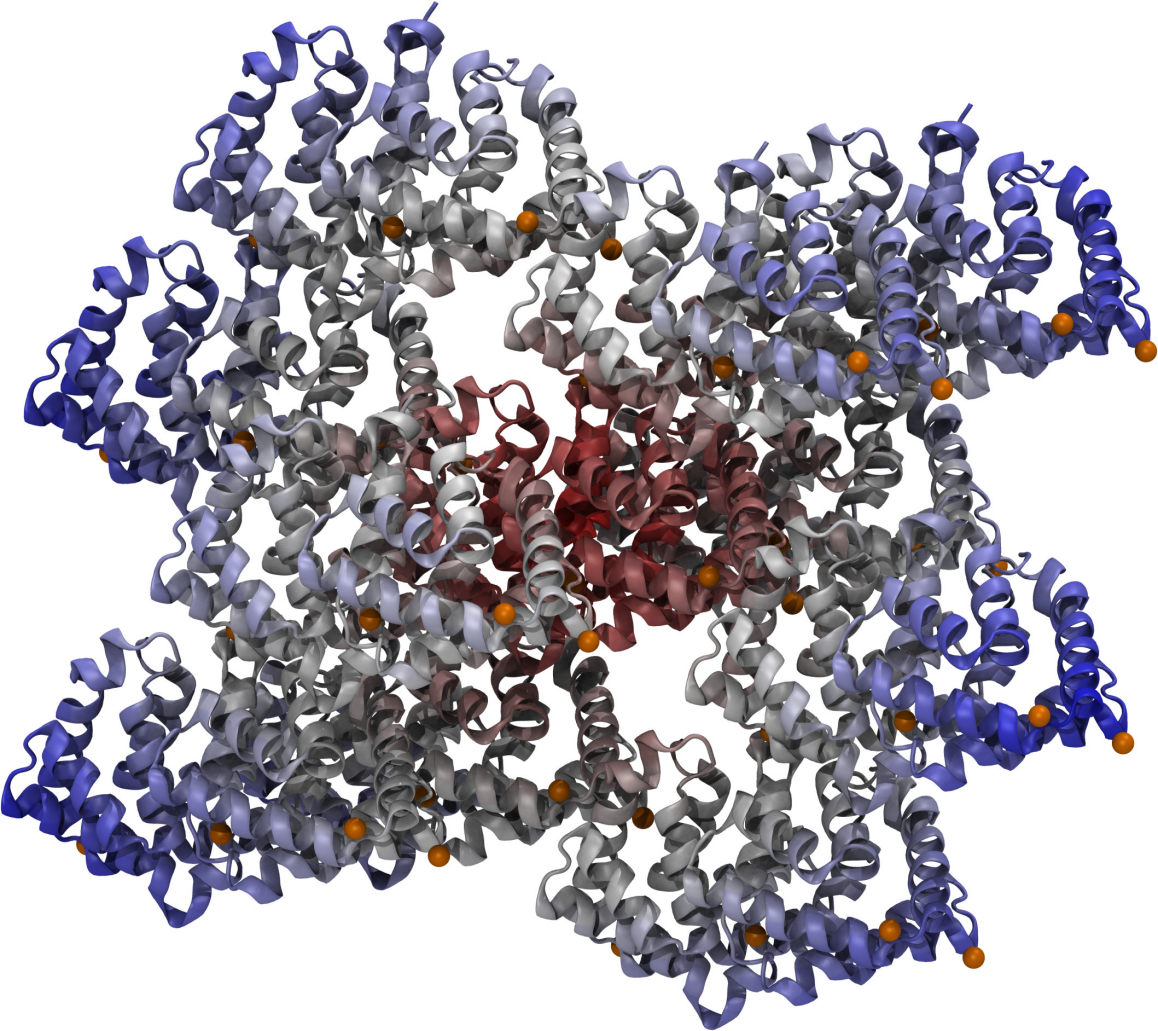
Fel d 1 in its crystallographic environment. Red depicts the central protein, while the white-to-blue gradient indicates surrounding protein molecules. Calcium ions are shown as orange spheres.

The three calcium binding sites proposed for Fel d 1 (Fig 1) were analyzed by semi-empirical calculations and molecular dynamics (MD) simulations. Three conditions were calculated semi empirically: with implicit solvent, without implicit solvent, and including crystallographic waters. The distance and bond orders obtained from these calculations (Table 1) indicate that the central Ca^2+^ is stable even if a little dislocated in its position, while the lateral ions are not bound and move from their proposed locations. MD simulations carried out in three force fields (AMBER, CHARMM, GROMOS) indicate the same dislodgment of the lateral Ca^2+^ ions (Table 2 and S2 Fig). To inspect if crystal packing effects would stabilize these ions, the neighboring asymmetric units from the crystallographic Fel d 1 were regenerated and simulated (Fig 3). There was no stabilization detected in this condition as well (Table 2).

**Table 1:** Distances and bond orders measured by semi-empirical calculation.

**Lateral Ca** ^**2+**^ **(A)**		**With implicit solvent**			**Without implicit solvent**			**With crystallographic waters**		
	**O atom**	**Distance (Å)**		**Bond order**	**Distance (Å)**	**Bond order**		**Distance (Å)**	**Bond order**	
Asp46A	Side chain	-		-	2.2	0.274		-	-	
	Main chain	2.2		0.234	-	-		-	-	
Met49A	Main chain	2.3		0.291	2.4	0.102^†^		2.4	0.094^†^	
Thr50A	Side chain	2.3		0.230	-	-		-	-	
Glu51A	Side chain	-		-	2.3	0.313		2.3	0.279	
	Side chain	-		-	-	-		2.3	0.243	
	Main chain	-		-	2.3	0.268		-	-	
H_2_O	Water 1	NA		NA	NA	NA		2.4	0.222	
	Water 2	NA		NA	NA	NA		2.4	0.207	
	Water 3	NA		NA	NA	NA		2.4	0.179^†^	
**Central Ca** ^**2+**^		**With implicit solvent**			**Without implicit solvent**			**With crystallographic waters**		
	**O atom**	**Distance (Å)**	**Bond order**		**Distance (Å)**		**Bond order**	**Distance (Å)**		**Bond order**
Asn89A	Side chain	2.2	0.255		2.3		0.238	2.3		0.208
Ile125A	Main chain	2.3	0.187^†^		2.5^‡^		0.086^†^	-		-
Asn89B	Side chain	2.3	0.195^†^		2.3		0.219	2.3		0.227
Ile125B	Main chain	-	-		2.3		0.182^†^	-		-
Asp130B	Side chain	2.3	0.248		2.3		0.268	2.3		0.278
	Side chain	2.3	0.257		2.3		0.268	2.3		0.227
H_2_O	Water 7	NA	NA		NA		NA	2.4		0.197^†^
	Water 8	NA	NA		NA		NA	2.5^‡^		0.122^†^ ^‘^
**Lateral Ca** ^**2+**^ **(B)**		**With implicit solvent**		**Without implicit solvent**		**With crystallographic waters**	
	**O atom**	**Distance (Å)**	**Bond order**	**Distance (Å)**	**Bond order**	**Distance (Å)**	**Bond order**
Asp46B	Side chain	-	-	2.3	0.192^†^	2.2	0.226
	Main chain	2.3	0.223	2.3	0.238	-	-
Ala47B	Main chain	2.3	0.211	-	-	-	-
Met49B	Main chain	2.3	0.264	2.4	0.148^†^	2.3	0.157^†^
Thr50B	Main chain	-	-	2.5^‡^	0.102^†^	-	-
Glu51B	Side chain	2.3	0.250	2.3	0.237	-	-
	Side chain	-	-	2.3	0.296	-	-
H_2_O	Water 4	NA	NA	NA	NA	2.3	0.250
	Water 5	NA	NA	NA	NA	2.3	0.301
	Water 6	NA	NA	NA	NA	2.3	0.261

^†^Bond orders of less than 0.2 are indicative of “no bond” [21]

^‡^Distances greater than reference distances for calcium coordination in proteins (2.35 Å-2.45 Å) [22].

**Table 2:** Average distances measured for molecular dynamics simulations under different force fields (Å).

	*	AMBER	CHARMM	GROMOS	Crystal	Expected*
Lateral Ca^**2+**^(A)	Asp46A	37.0±1.0	11.1±1.5	33.7±13.3	13.5±14.8	2.4
	Met49A	36.8±8.9	10.0±1.3	37.3±13.7	14.2±15.4	2.3
Central Ca^**2+**^	Asn89A	2.7±0.1	2.3±0.1	2.5±0.3	2.4±0.1	2.4
	Ile125A	2.7±0.1	4.3±0.7	7.2±0.8	4.2±1.0	2.2
	Asn89B	2.7±0.3	5.3±1.0	4.9±0.6	4.2±0.8	2.7
	Asp130B	2.6±0.1	2.3±0.3	2.7±0.2	5.2±0.2	2.2
Lateral Ca^**2+**^(B)	Asp46B	4.1±0.9	16.1±1.8	27.3±11.2	7.6±4.5	2.4
	Met49B	5.3±0.6	13.2±2.1	27.0±11.0	6.7±3.7	2.3

*residues making contact with calcium ions and their expected distances were taken from PDB ID 2EJN [8].

### Calcium effects on protein conformation

Considering that the stable binding of the central Ca^2+^ could be related to stabilization of the protein dimer, the interaction energy of the complex was also measured for the performed MD simulations (Table 3 and S3 Fig). There was a slight decrease in interaction energy (more favored interaction) in the calcium-free conditions when compared to calcium-bound conditions.

**Table 3:** Interaction energies measured between Fel d 1 monomers during molecular dynamics simulations under different force fields (kJ/mol).

	AMBER	CHARMM	GROMOS
With Ca^**2+**^ ions	-718.07±65.49	-599.44±69.71	-570.57±67.84
Without Ca^**2+**^ions	-728.87±69.52	-772.41±67.47	-617.71±87.07

**Fig 4:**
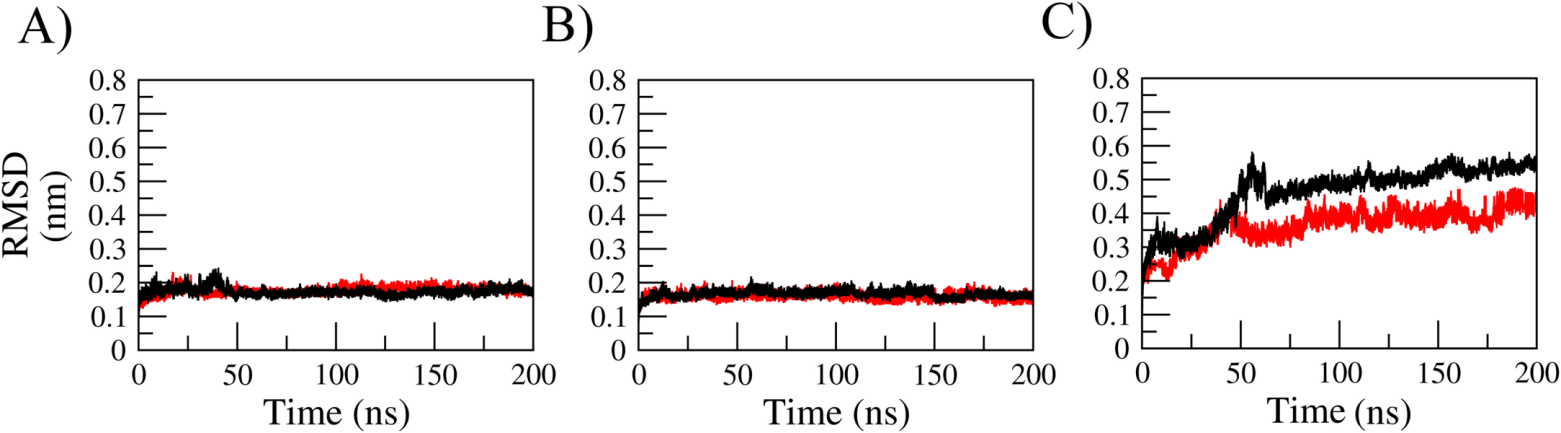
All-atom root mean square deviation (RMSD) measured for different force field simulations of Fel d 1. (A) AMBER, (B) CHARMM, (C) GROMOS. Color coding: Simulations without Ca^**2+**^ions (black), simulations with Ca^**2+**^ ions (red).

**Fig 5:**
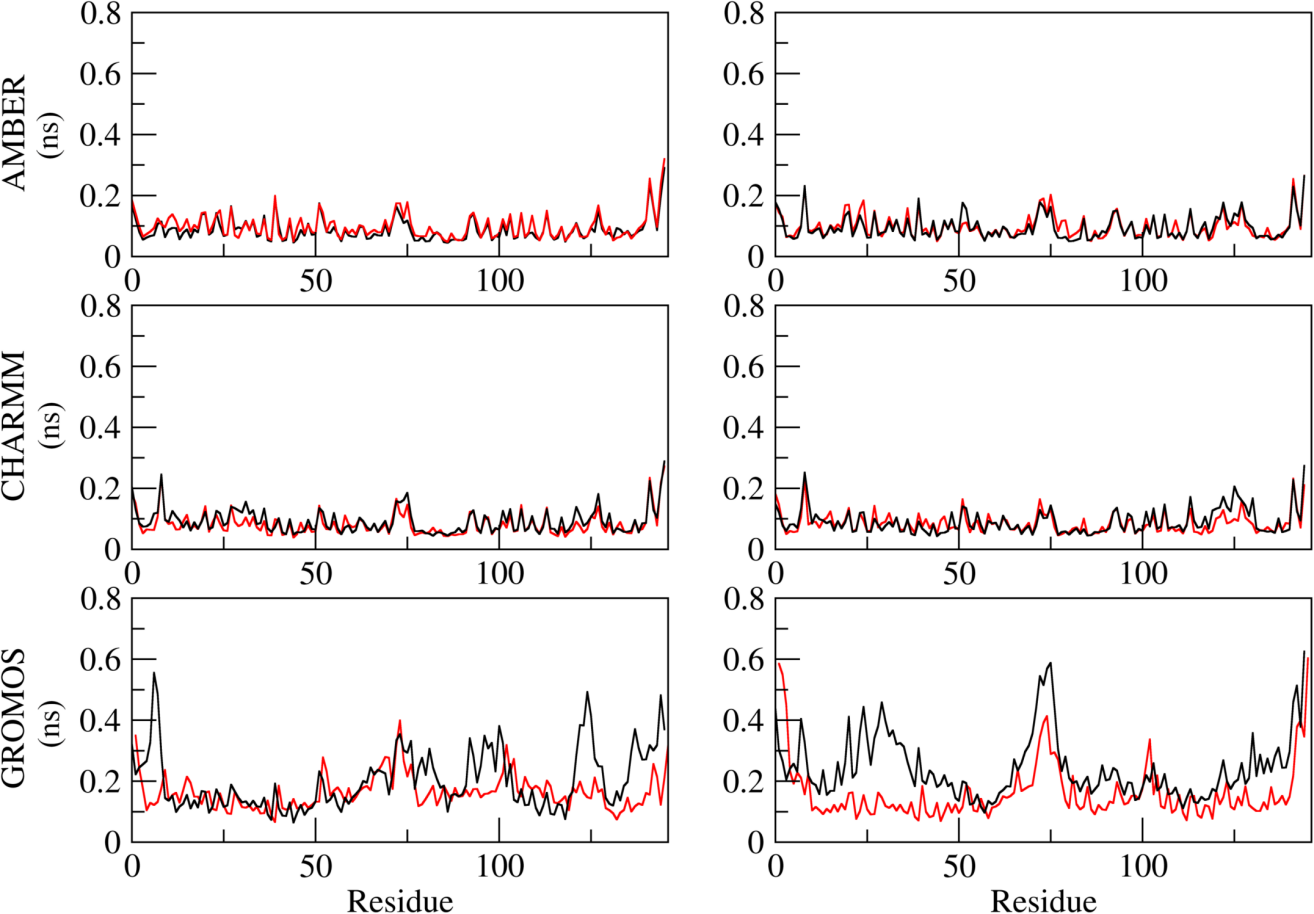
Root mean square fluctuations (RMSF) measured for different force field simulations of Fel d 1. Color coding: Simulations without Ca^**+2**^ ions (black), simulations with Ca^**+2**^ ions (red).

The MD simulated systems were generally stable (Fig 4) and had no difference regarding their stability with or without Ca^2+^ for simulations under AMBER and CHARMM force fields. In the simulations performed under GROMOS force field, however, there was a slight difference between metal-bound and metal free conditions combined with noticeable difference in stability in comparison with the other force fields. (Fig 4). Regarding local flexibility, we observed little differences between calcium-free and calcium-bound system (Fig 5).

### Glycosylation effects

**Fig 6:**
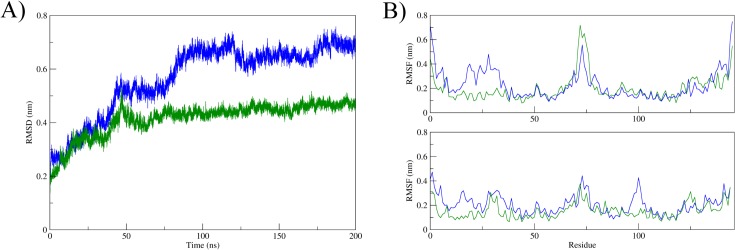
Structural flexibility of glycosylated Fel d 1. (A) All-atom root mean square deviation (RMSD) and (B) Root mean square fluctuations (RMSF) measured for the simulations with minimal (blue) and full glycosylations (green).

The minimally and fully glycosylated systems behaved differently. While the fully glycosylated protein had a conformational behavior equivalent to the unglycosylated forms in the same force field (GROMOS), the protein with minimal glycosylation had a greater divergence in terms of conformation (Fig 6).

Moreover, glycosylation seemed to increase flexibility around residue 75, in a loop region of chain A (but not on the equivalent region of chain B) (Fig 6). The minimal saccharide structure also caused an increase in flexibility in the N-terminus region of both chains. The secondary structure content was similar in all simulations, with a noted decrease in helical content for the systems with no Ca^2+^ and with the minimally glycosylated protein (S4 Fig). This decrease was associated with partial unfolding of helices I and II (first and second helices from the N-terminus), reflected in the increased flexibility observed in RMSF analyses (Fig 6). The analysis of secondary structure along the simulated time also indicates structural stability for (at least) the last 20 ns of all simulation with the GROMOS force field. It is interesting to note that the full glycosylation seemed to stabilize the Chain B structure, as revealed by RMSD analyses of each of the protein segments (half-monomers in the fused protein) (S5 Fig). This protein-segment analysis also confirmed the early stabilization of all chains in the Ca^2+^ system.

### Cavity assessment and ligand dockings

The two cavities detected in Fel d 1, which had different volumes in the crystallographic structure [8] were measured for all systems simulated with GROMOS force field. Their volume seems to oscillate periodically along the simulated time (S6 Fig). This is especially clear by analyzing the three main structural clusters found in each simulation (Table 4). The cavities detected in these clusters confirm clearly different cavity sizes for each Fel d 1 chain.

**Table 4:** Cavity volume measured for the three main structural clusters from MD simulations under different conditions.

		Volume (Å³)		
		Cluster 1	Cluster 2	Cluster 3
With Ca^2+^	Chain A	4357	4133	4174
	Chain B	5145	4004	3783
Without Ca^2+^	Chain A	4397	4590	3208
	Chain B	4035	4383	4454
Glycosylated (minimal)	Chain A	3133	3537	3634
	Chain B	2907	4367	4217
Glycosylated (full)	Chain A	3462	4032	3344
	Chain B	4607	4031	3549

**Fig 7:**
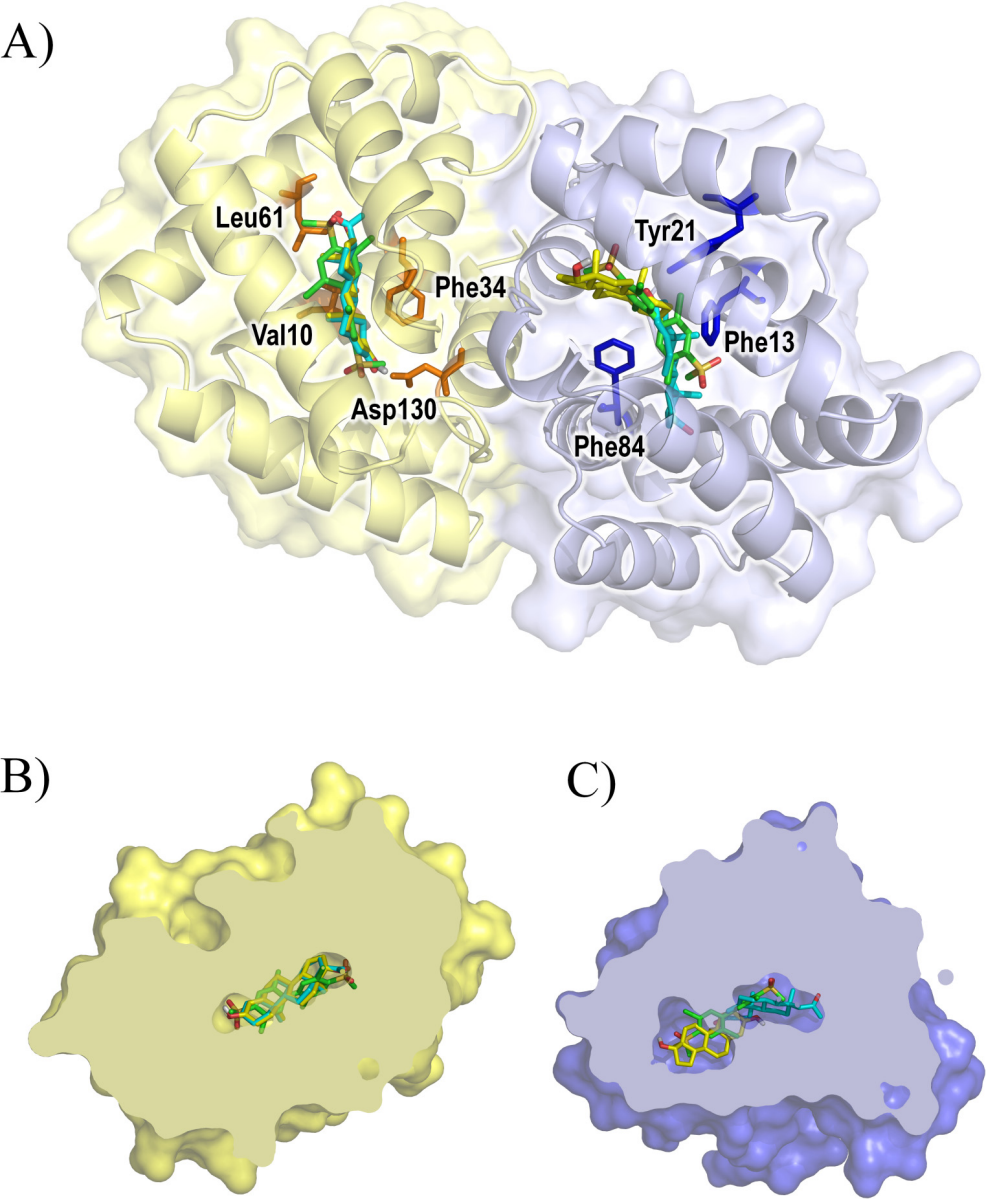
Docking of different ligands to Fel d 1. (A) Docking results overview, highlighting residues involved in binding interactions; Cut-through views of (B) chain A and (C) chain B, highlighting differences in cavity sizes. Cut-through views are rotated in respect to (A) for clarity; chain A shown in yellow and chain B shown in blue, testosterone shown in yellow, progesterone shown in cyan, PCB shown in green.

We studied Fel d 1 cavity binding capacity using molecular docking, revealing that cavities of both Fel d 1 chains (A and B) are able to interact with the ligands PCB (an steroid analog), progesterone and testosterone (Fig 7). In chain A, residues Val10, Leu61 and Phe84 presented hydrophobic interactions with all three ligands, and Asp130 was responsible for one hydrogen bond. In chain B, for all ligands, residues Phe13 and Phe84 were the ones making the hydrophobic contacts and Tyr21 was the residue responsible for one hydrogen bond.

### Dynamical network and motion analyzes

**Fig 8:**
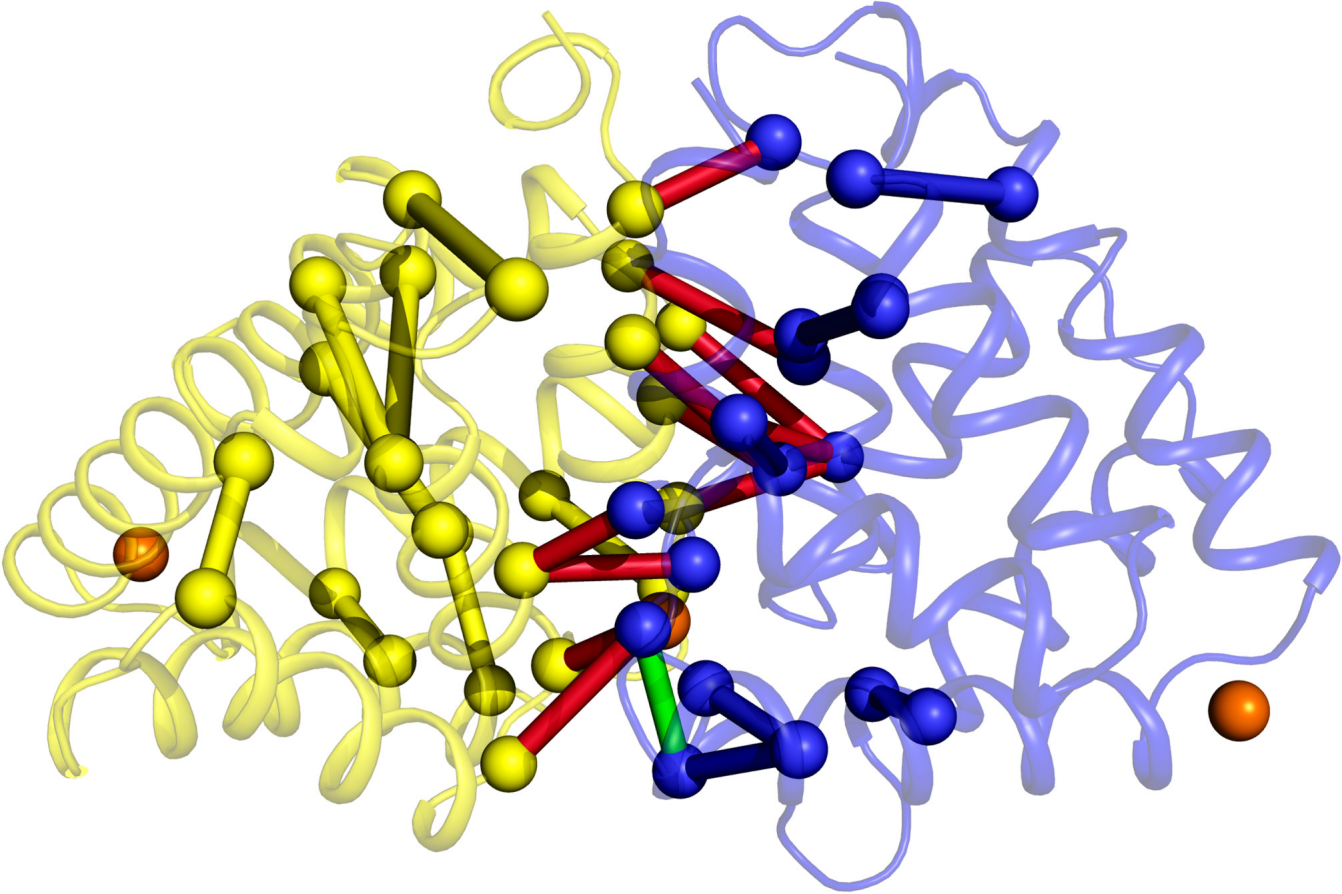
Overview of essential node pairs for motion transmission in Fel d 1 simulations. ‘Hinge’ pairs in each chain are colored yellow for Chain A and blue for Chain B, while interface pairs are colored red. A connection between interface and intrachain motions (detected in the system simulated with Ca^**2+**^ and no glycosylation) is shown in green. Calcium ions shown in orange.

Inspecting internal motion correlations for the simulated Fel d 1 systems with GROMOS force field, we were able to detect two major trends. First, there is conservation of residues acting as critical nodes for motion transmission between chains A and B at the interaction interface, regardless of the presence of calcium or glycosylation (S2 Table). Second, there seems to be a hinge-like motion transfer between each original chain (i.e. each half of Fel d 1 crystallographic monomer) in the simulated systems (Fig 8). The observed critical nodes, also conserved, seem to act as unconventional hinges, and an oscillatory motion between these ‘flaps’ can be observed, especially for Chain A (S7 Fig), although the characteristic disulfide bridges of Fel d 1 may restrict this oscillation.

**Fig 9:**
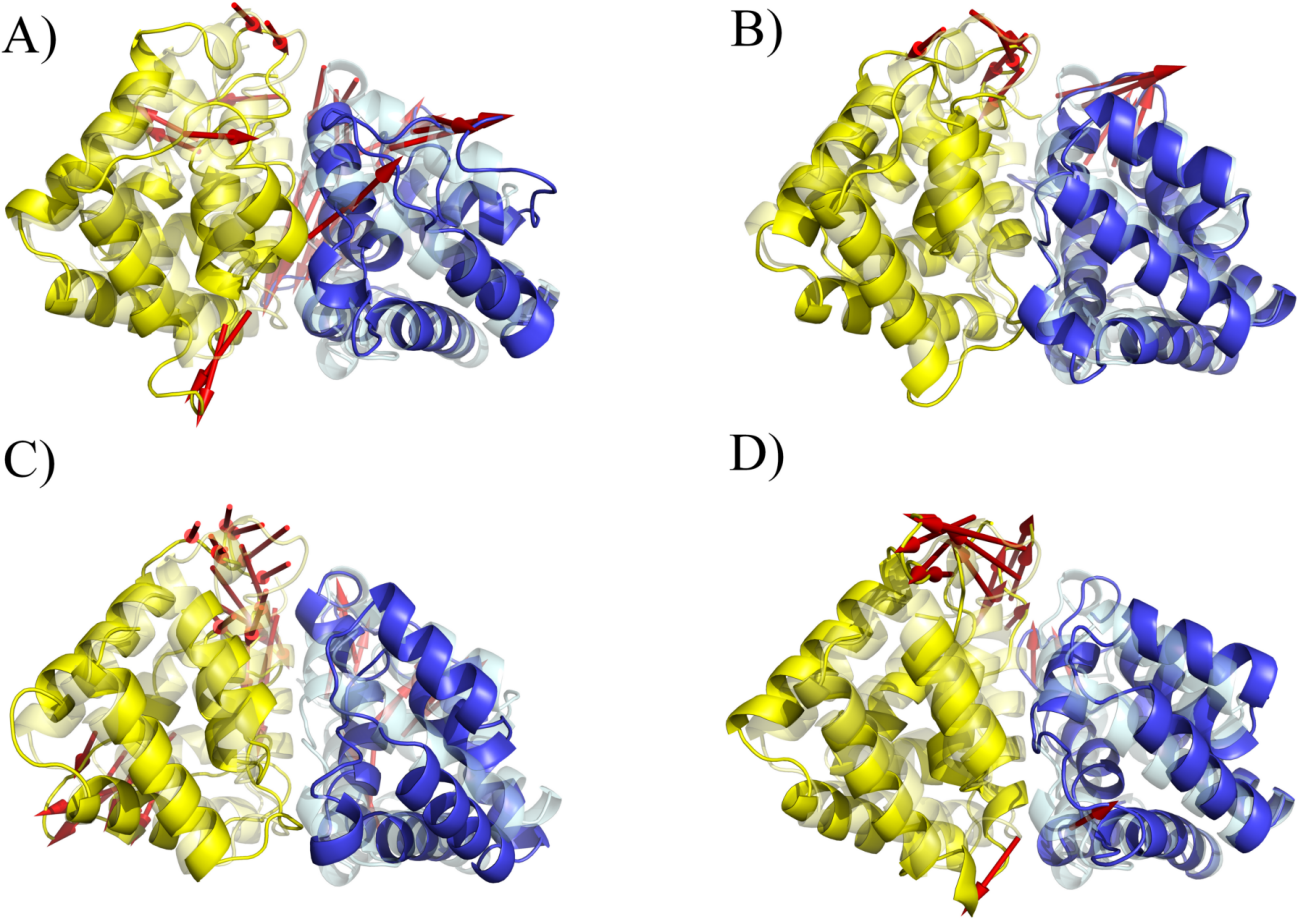
Overview of conformational changes observed for the different GROMOS simulated systems. The initial structures are shown as transparent, with arrows pointing the major motions observed between the initial and final stages of the MD simulations. (A) Calcium-free system; (B) Calcium-bound system; (C) Minimally glycosylated system; (D) Fully glycosylated system. Chain A shown in yellow and chain B shown in blue.

The motions of each protein chain in respect to each other and within each chain (Fig 9) reveal no clear pattern. The calcium-free system has diffuse motions, whereas the other systems seem to have more directed movements. This is especially highlighted in the glycosylated systems, in which the chains of Fel d 1 (monomers in the fused protein) seem to move in the opposite direction of each other. Helices I and II show pronounced mobility (in chain A).

## Discussion

Considering the debate on whether Ca^2+^ binds to Fel d 1 or not, for this work we employed different calculation approaches to inspect such interactions. Our results indicate that the central ion is bound to the protein, while the lateral ions are free. This may hinder the proposed action of Fel d 1 in allergy through modulation of phospholipase A_2_ activity via calcium sequestration [8, 10]. This property was proposed taking uteroglobin as a model, but even for this protein there is controversy regarding the putative binding of the ion [11–13].

Supporting the concept that Ca^2+^ is not bound to the lateral sites of Fel d 1 is the physiological concentration of calcium in cats. Among the synthesis sites of Fel d 1 (which include perianal, sebaceous, and salivary glands), the calcium concentration in submandibular salivary glands (20 mmol/kg) is considered high for soft tissues in mammals (twice as much as normal human lung tissue) [23, 24], but the calcium concentration in the crystallization medium was 25-fold greater than that [8]. This would lead to unspecific binding of Ca^2+^, and a fourth ion in the final crystallized structure is considered one of these cases [8].

To avoid possible parameter bias in simulating this protein-metal complex, we used three different force fields. There were differences in overall flexibility observed between all-atom force fields (AMBER and CHARMM) and united-atoms force field (GROMOS) in this work. These differences, which may be striking at first, are probably due to faster convergence and enhanced conformational sampling in the united-atoms force field [25]. The alternative explanation to GROMOS apparently not reaching convergence is that AMBER and CHARMM simulations remain stuck in energetic wells in a more rugged energy surface than in GROMOS simulations. However, the concept of convergence involves evaluating if what has been measured is enough to discard the occurrence of anything important which remains unmeasured [26]. There is no guarantee that a stable system (based on the generally used measures, such as RMSD) will remain stable if simulated for some extra nanoseconds. Besides that, convergence varies with every property analyzed and they do not correlate to each other [27]. It has been shown that evaluating convergence is a subjective matter, and that the evaluation of structural features such as RMSD for this purpose is very unreliable [28]. In the context of our work, they show that different levels of detail in the force field parametrization have no difference in describing the Ca^2+^ behavior, allowing us to compare the GROMOS results obtained for the calcium ion studies with the glycosylation studies with the same force-field, for which there is a wealth of saccharide parameters [18,19]. Besides the combination of different simulations, including the crystal environment, the semi-empirical calculations improved the evaluation of such important aspect of Fel d 1 structural biology by exploring the electronic component of Ca^2+^ ions coordination in this complex. It is possible that Ca2+ ions can bind to different, new sites on Fel d 1, based on the behavior of the GROMOS simulations. This observation reinforces that, apart from the central ion binding site, the lateral binding sites are not stable and are not supported by different inspections.

Another highlighted feature of the major cat allergen is its saccharide content. The active form of Fel d 1 has two N-glycosylation sites (Fig 2), and the attached glycan chains are highly variable [14]. For this work we inspected the largest and the smallest carbohydrate chains observed linked to the protein. Different effects were observed in each case. While the conformational behavior of the fully glycosylated protein did not differ from its unglycosylated counterpart in general, the minimally glycosylated protein behaved differently. There was an increase in flexibility, due to partial unfolding of two helices of one of the Fel subunits. The fully glycosylated protein, however, presented a reduction in its cavity size, also for one of its subunits. This difference could be due to sampling limitations in our simulations, but an extension of differences between subunits detected in the crystallographic structure [8] are not discarded.

A previous study of circular dichroism on Fel d 1observed decrease in its helical content due to removal of glycan portions [9]. In our work, we were able to pinpoint the helices that are more likely to unfold, corresponding to this previous observation. The reduction in cavity volume observed for one subunit of the fully glycosylated protein may hint to Fel d 1glycans acting on molecular transport modulation. It has been shown previously that selective saccharide removal from glycans in human plasmatic transport proteins caused cavity alterations, affecting its binding do hormones and drugs [29].

The putative content of Fel d 1 cavities has not been identified yet [8]. A similar protein, found in the slow loris (*Nycticebus coucang*, a prosimian), is capable of causing anaphylactic shock in humans [30]. These animals are thought to use these proteins defensively, but mainly as a communication tool. Different hydrophobic compounds were identified in association with this molecule, and it is proposed that the protein structure would act as a box, containing and delivering chemical messages [31]. It has been observed that cat females secrete less Fel d 1 than males, and that males that are handling-avoidant (less frightened and more aggressive) have greater release of the allergen than handling-friendly males [32]. This may be an indication of Fel d 1 acting in information transmission in cats.

Considering the synthesis of the protein in perianal glands and its spreading by grooming involving saliva, it is tempting to propose that steroid-like molecules from the perianal glands may be entrapped in Fel d 1 by changes in pH (or other feature) brought about by interaction with saliva. Based on the crystal structure of uteroglobin bound to an steroid analog [33], we performed dockings and confirmed that the Fel d 1 cavities observed in simulation would accommodate progesterone (312 Å^3^), testosterone (399 Å^3^), and the steroid analog, PCB (335.6 Å^3^). A communication role has been proposed for the loris brachial gland protein, (a Fel d 1 fold analog) [27], and has also been proposed for Fel d 1 in the 1990s [34].

Fel d 1 is a dimer of heterodimers in its native form, and was expressed as a dimer of fused monomers for crystallization [8]. For this reason, each monomer in the crystal (originally a dimer of chains α and β) is called a chain (A and B). Despite being a homodimer, the interacting interface of the oligomer is not symmetric. Asymmetry is important for the proper recognition of each monomer (α and β dimer) in terms of immunological response [15]. The relevance of asymmetry for the biological action of the Fel d 1 dimer (or dimer of dimers in its native form) has not been investigated so far, but our results point to differential effects of glycosylation and cavity behavior in each of the asymmetrically positioned Fel d 1 subunits.

In summary, in this work we were able to apply theoretical chemical tools in the study of Fel d 1 molecular behavior and its interaction with calcium ions and glycosylation. Our results not only corroborate previous observations, but also widen the breadth of action of the major cat allergen. We hope to have highlighted some advantages of the computational structure biology applied to the study of Fel d 1. Future investigations, focused on the influence of variable glycosylation structures on the protein, site-directed mutagenesis of metal-binding and motion-transmitting residues, and especially those focused on the functions carried out by Fel d 1 in feline physiology, may benefit from the observations presented here. For instance, the calcium-binding properties of the protein are less prominent than previously proposed, reducing the ion structural role. The protein binding cavity is compatible with previously proposed ligands, providing structural basis for these proposals. The impact of glycosylation on Fel d 1 seems limited, also reducing its importance for cavity volume control. The models presented here provide structural and rational basis to guide future studies with ligand binding and site-directed mutagenesis. We hope this kind of work may highlight the need for cooperation between laboratory experimentalists and computer modelers with the joint aim of studying structural biology in atomistic scale in both time and space dimensions.

## Materials and Methods

### Simulated systems

We retrieved the structure for the tetrameric form of Fel d 1 from the RCSB Protein Data Bank under PDB ID 2EJN [8], and used it to prepare nine Fel d 1 systems for MD simulations under different force fields and conditions. The crystal-derived Fel d 1 was simulated with and without crystallographic Ca^2+^ ions employing three force fields (AMBER99SB-ILDN [35], CHARMM27 [36], and GROMOS53a6 [20]). Two glycosylated forms of the protein were also simulated, as derived from MALDI-MS analyses of the native Fel d 1, basically corresponding to a desialylated (minimal or smallest) and a sialylated (full or largest) oligosaccharide structures [14]. We also regenerated the crystalline environment surrounding the tetrameric protein using symmetry tools from PyMol (Schrödinger, LLC) and symmetry group information from the Fel d 1 crystal. This procedure added ten other tetramers to this ninth simulated system.

### Glycoprotein construction

Each dimer of Fel d 1 has one N-glycosylation site, at Asn103 [8], in a total of two sites for the biological tetramer [14]. We used the full length [(-Asn-) GlcNAc_2_Man_3_GlcNAc_3_Gal_3_ (Fuc_1_)-NeuAc_3_] and truncated [(-Asn-) GlcNAc_2_Man_3_GlcNAc_3_Gal_2_] forms of the glycosidic chains (named ‘full’ and ‘minimal’ for reference) previously identified [14] to build the glycosylated proteins. This construction was made via Glycosciences.de modelling tools [37, 38]. The glycosidic linkages composing such glycans had their geometries adjusted to each of their relative abundance as isolated disaccharides in water, thus determined as their main conformational states [39, 40]. Such procedure was successfully applied in previous glycoprotein studies [41, 42]. The oligosaccharide topologies were described under GROMOS 53A6glyc parameters set [18, 19] and the protein moieties under the standard GROMOS96 53A6 force field.

### Molecular dynamics studies

We submitted the Fel d 1 systems to MD simulations with the GROMACS 4.5 suite [42] for 200 ns each, with the exception of crystal-environment mimic, which we simulated for 25 ns. The systems were solvated in rectangular boxes using periodic boundary conditions, and subjected to different force fields and water models: GROMOS96 53a6 force field [20] with SPC water models [43], CHARMM27 [32] with TIP3P [44], and AMBER99SB-ILDN [31] also with TIP3P. The Lincs method [45] was applied to constrain covalent bond lengths, allowing an integration step of 2 fs after an initial energy minimization using Steepest Descents algorithm. Electrostatic interactions were calculated with Particle Mesh Ewald method [46], in which short-range interactions cutoff values were set to 0.9 nm. Temperature and pressure were kept constant by coupling proteins, ions, and solvent to external temperature and pressure baths with coupling constants of τ = 0.1 and 0.5 ps (Berendsen barostat, velocity rescaling thermostat [47, 48], respectively). The dielectric constant was treated as ε = 1, and the reference temperature was adjusted to 300 K to reflect the temperature faced by the protein in cat hair and dandruff. The systems were slowly heated from 50 to 300 K, in steps of 5ps, each step increasing the reference temperature by 50 K, allowing a progressive equilibration of the molecular system. The simulations were performed with no restraint, with a reference value of 3.5 Å between heavy atoms for a hydrogen bond, and a cutoff angle of 30° between hydrogen-donor-acceptor [41]. The so called “crystal-environment mimic” was based on reproducing the crystallographic Fel d 1 neighboring asymmetric units only, not their entire crystallographic chemical environment. The system sizes for all simulations are listed on S3 Table.

Cavities were measured with Mole2 [49, 50]. Essential nodes for motion transmission were inspected as described previously for tRNAs [51, 52]. Ligand volumes were calculated with UCSF Chimera 1.7 [53]. Docking studies of Fel d 1 chains A and B to ligands PCB, progesterone and testosterone were each performed with Autodock 4.2 [54] and lowest energy conformations for each system were analyzed with Poseview [55] and Ligplot [56]. All other analyses were carried out with dedicated tools from the Gromacs suite.

### Semiempirical calculations

In order to add information on metal coordination to molecular mechanics calculations we submitted Fel d 1 with all four crystallographic Ca^2+^ ions to semiempirical quantum chemistry calculations using MOPAC [57] with the PM6 Hamiltonian [58] and MOZYME linear scaling method [59]. The following steps, based on a previous study of metal coordination [60], were employed on the calculations: 1) hydrogen atoms optimization to a gradient lower than 5 kJ/mol/Å; 2) global optimization with gradient convergence criterium of 10 kJ/mol/Åand cutoff = 6; 3) global optimization with gradient convergence criterium of 5 kJ/mol/Åand cutoff = 9. We carried out calculations in the absence of crystallographic water molecules, with the COSMO continuum solvation model [61], and with all the crystallographic water molecules within a 5 Å radius from the Ca^2+^ ions. We used the obtained minimum energy conformation was employed to evaluate the bond orders and distances to estimate the possible coordination between amino acid residues and Ca^2+^ ions.

## Supporting Information

S1 Fig Validation of GROMOS 53A6glyc parameters implemented for sialic acid. (PDF)

S2 Fig Distances measured for molecular dynamics simulations of Fel d 1 under different force fields. Please refer to Table 1 for details. (PDF)

S3 Fig Interaction energies measured between Fel d 1 monomers during molecular dynamics simulations under different force fields. Color coding: Simulations without Ca^2+^ ions (black), simulations with Ca^2+^ ions (red).
(PDF)

S4 Fig Secondary structure content for the Fel d 1 systems simulated with GROMOS. (A) Fel d 1 without Ca^2+^; (B) Fel d 1 with Ca^2+^; (C) Fel d 1 with minimal glycosylation; (D) Fel d 1 with full glycosylation.
(PDF)

S5 Fig All-atom root mean square deviation (RMSD) measured for Fel d 1 segments corresponding to the original chain regions (fused in the crystallized protein). (A) Calcium-free system; (B) Calcium-bound system; (C) Minimally glycosylated system; (D) Fully glycosylated system. Region A_1_ in black, region A_2_ in red, region B_1_ in blue, region B_2_ in green.
(PDF)

S6 Fig Cavity volume overview (calcium-free system as an example). (A) initial cavity volume, (B) final cavity volume (cavity volumes shown as green surfaces); (C) cavity volume measurements for each Fel d 1 chain in four simulations.
(PDF)

S7 Fig Structural ‘oscillation’ observed for (A) chain A, and (B) chain B of Fel d 1 in four different simulations. The distance between each original chain in the native Fel d 1 (half chains of the crystallographic protein) is shown. The oscillation may indicate an opening-closing behavior. (calcium-free system in black, calcium-bound system in red, minimally glycosylated system in blue, fully glycosylated system in green; distances measured for the centers of mass in each structure).
(PDF)

S1 Table Parameters used in the GROMOS 53A6glyc force field for sialic acid, in a GROMACS-compatible format. These encompass atom types, atomic partial charges, charge-group definition, bond stretching, bond-angle bending, improper dihedral deformation and torsional potential dihedral.
(PDF)

S2 Table Essential node pairs detected in four Fel d 1 simulated systems. Nodes that appear more than once are grouped by color. Interactions detected between pairs by PDBsum for the crystallographic structure are in parentheses.
(PDF)

S3 Table Sizes of simulated systems. (PDF)
