# Supplementary figures and images for: The Calcium Goes Meow: Effects of Ions and Glycosylation on Fel d 1, the Major Cat Allergen
Source: PLoS One. 2015 Jul 2;10(7):e0132311. doi: 10.1371/journal.pone.0132311 (PMC4489793; doi:10.1371/journal.pone.0132311)

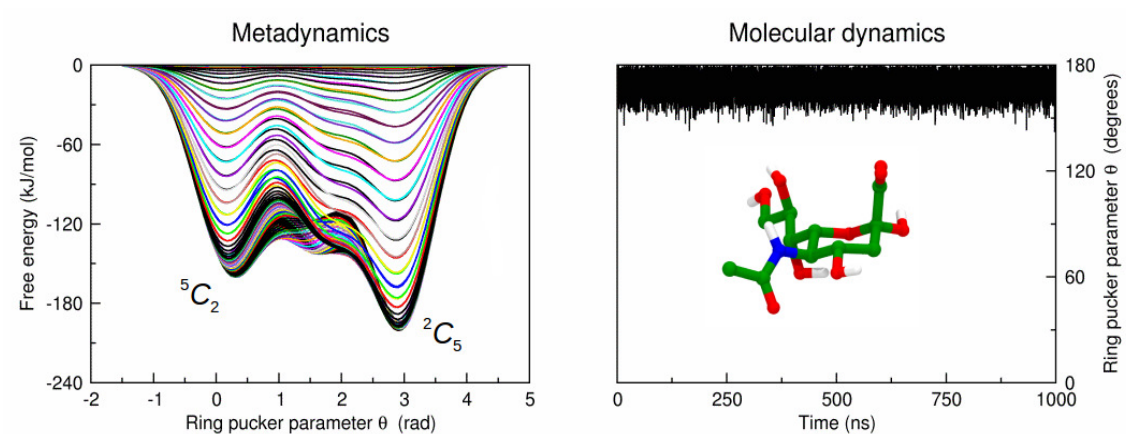

**Fig. S1. Validation of GROMOS 53A6glyc parameters implemented for sialic acid.**

Supplement: S1 Fig — (PDF) [file pone.0132311.s001.pdf]
