# Supplementary material for: The Calcium Goes Meow: Effects of Ions and Glycosylation on Fel d 1, the Major Cat Allergen
Source: PLoS One. 2015 Jul 2;10(7):e0132311. doi: 10.1371/journal.pone.0132311 (PMC4489793; doi:10.1371/journal.pone.0132311)
Supplement: S2 Fig — (PDF) [file pone.0132311.s002.pdf]

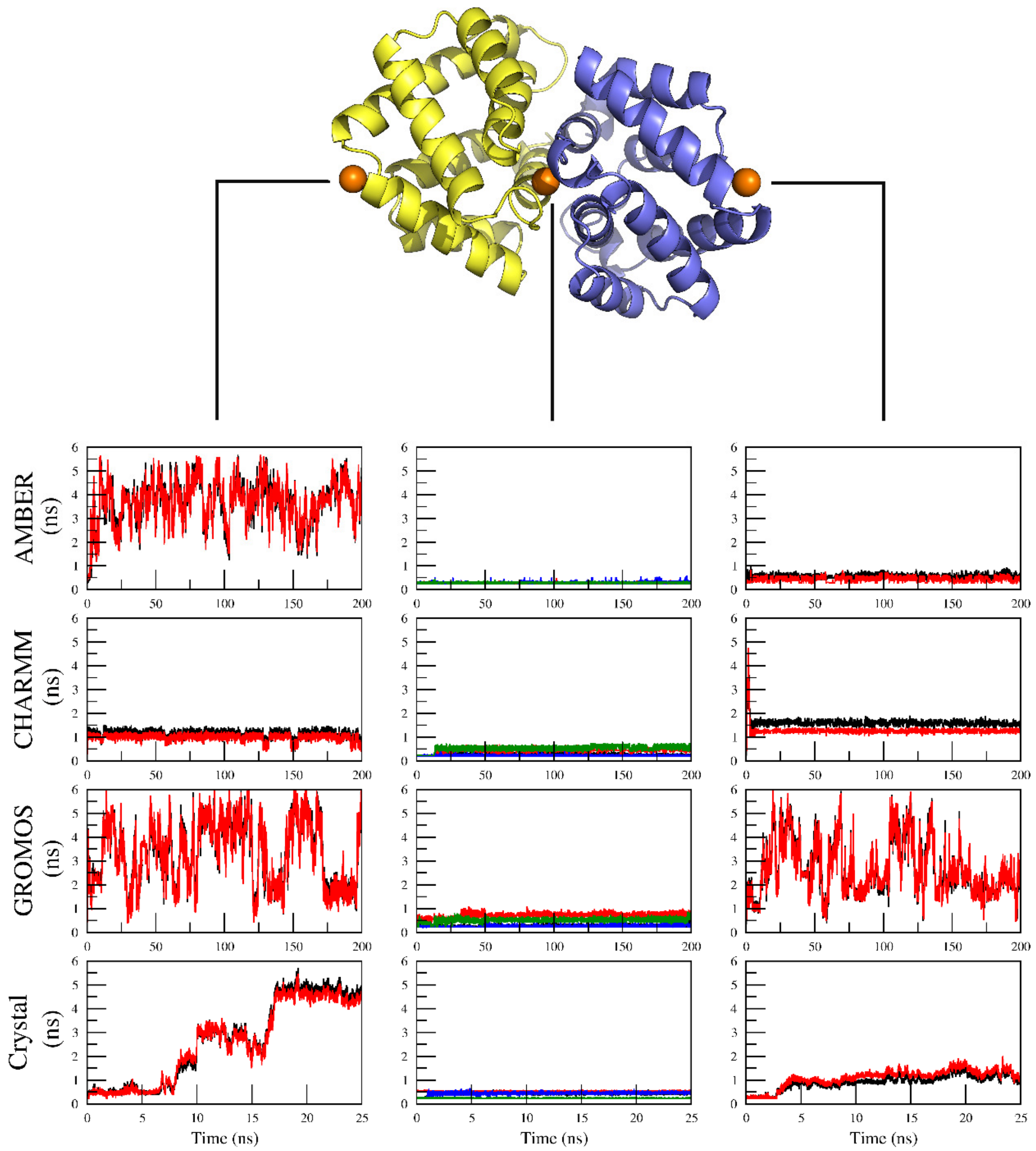

Fig. S2. Distances measured for molecular dynamics simulations of Fel d 1 under different force fields. Please refer to Table 1 for details.
