# Supplementary material for: The Calcium Goes Meow: Effects of Ions and Glycosylation on Fel d 1, the Major Cat Allergen
Source: PLoS One. 2015 Jul 2;10(7):e0132311. doi: 10.1371/journal.pone.0132311 (PMC4489793; doi:10.1371/journal.pone.0132311)
Supplement: S3 Fig — Color coding: Simulations without Ca2+ ions (black), simulations with Ca2+ ions (red). (PDF) [file pone.0132311.s003.pdf]

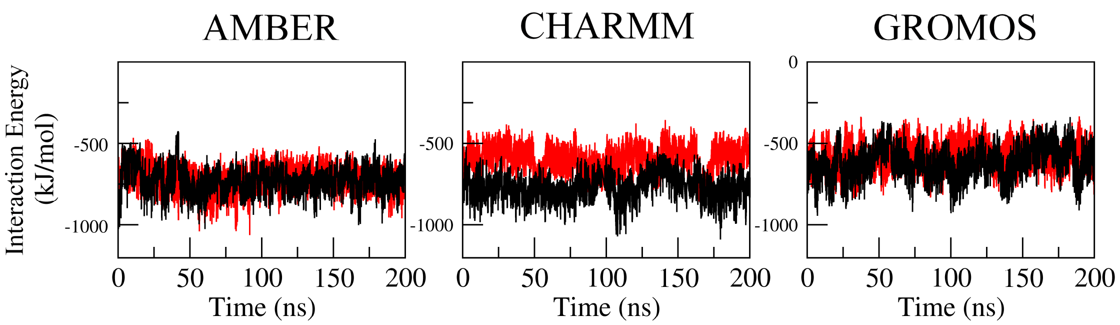

Fig. S3. Interaction energies measured between Fel d 1 monomers during molecular dynamics simulations under different force fields. Color coding: Simulations without Ca^{2+}ions (black), simulations with Ca^{2+} ions (red).
