# Supplementary material for: The Calcium Goes Meow: Effects of Ions and Glycosylation on Fel d 1, the Major Cat Allergen
Source: PLoS One. 2015 Jul 2;10(7):e0132311. doi: 10.1371/journal.pone.0132311 (PMC4489793; doi:10.1371/journal.pone.0132311)
Supplement: S4 Fig — (A) Fel d 1 without Ca2+; (B) Fel d 1 with Ca2+; (C) Fel d 1 with minimal glycosylation; (D) Fel d 1 with full glycosylation. (PDF) [file pone.0132311.s004.pdf]

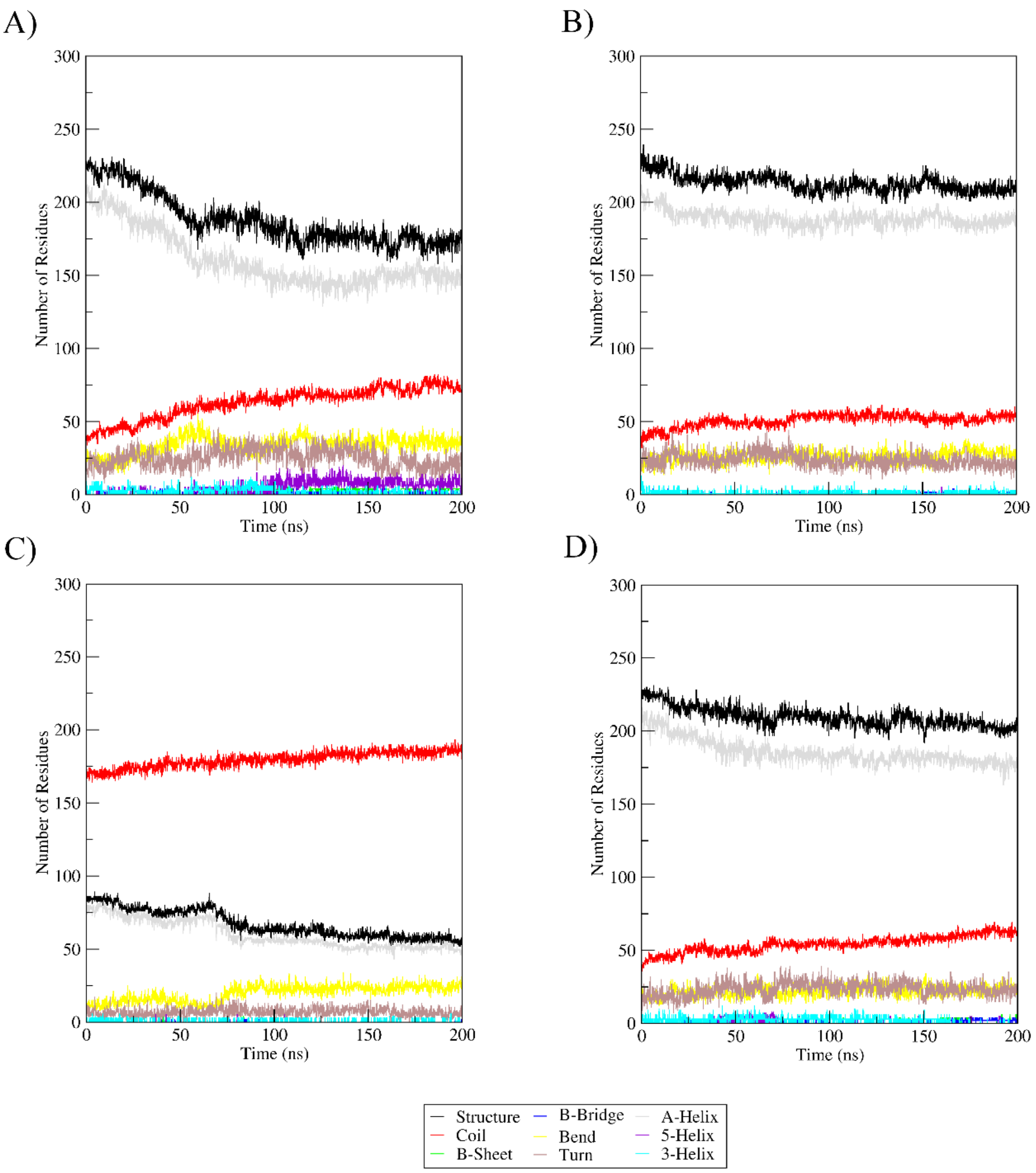

Fig. S4. Secondary structure content for the Fel d 1 systems simulated with GROMOS. (A) Fel d 1 without Ca^{2+}; (B) Fel d 1 with Ca^{2+}; (C) Fel d 1 with minimal glycosylation; (D) Fel d 1 with full glycosylation.
