# Supplementary material for: The Calcium Goes Meow: Effects of Ions and Glycosylation on Fel d 1, the Major Cat Allergen
Source: PLoS One. 2015 Jul 2;10(7):e0132311. doi: 10.1371/journal.pone.0132311 (PMC4489793; doi:10.1371/journal.pone.0132311)
Supplement: S5 Fig — (A) Calcium-free system; (B) Calcium-bound system; (C) Minimally glycosylated system; (D) Fully glycosylated system. Region A1 in black, region A2 in red, region B1 in blue, region B2 in green. (PDF) [file pone.0132311.s005.pdf]

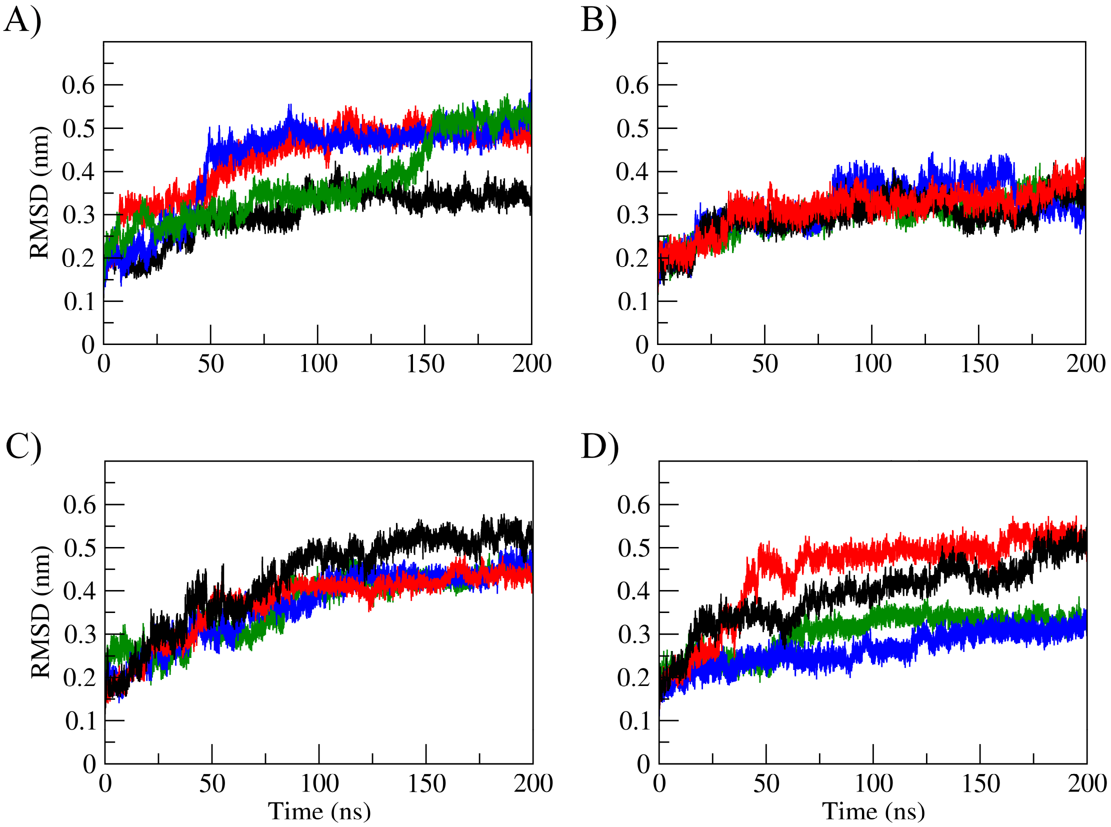

Fig. S5. All-atom root mean square deviation (RMSD) measured for Fel d 1 segments corresponding to the original chain regions (fused in the crystallized protein). (A) Calcium-free system; (B) Calcium-bound system; (C) Minimally glycosylated system; (D) Fully glycosylated system. Region A_1 in black, region A_2 in red, region B_1 in blue, region B_2 in green.
