# Supplementary material for: The Calcium Goes Meow: Effects of Ions and Glycosylation on Fel d 1, the Major Cat Allergen
Source: PLoS One. 2015 Jul 2;10(7):e0132311. doi: 10.1371/journal.pone.0132311 (PMC4489793; doi:10.1371/journal.pone.0132311)
Supplement: S6 Fig — (A) initial cavity volume, (B) final cavity volume (cavity volumes shown as green surfaces); (C) cavity volume measurements for each Fel d 1 chain in four simulations. (PDF) [file pone.0132311.s006.pdf]

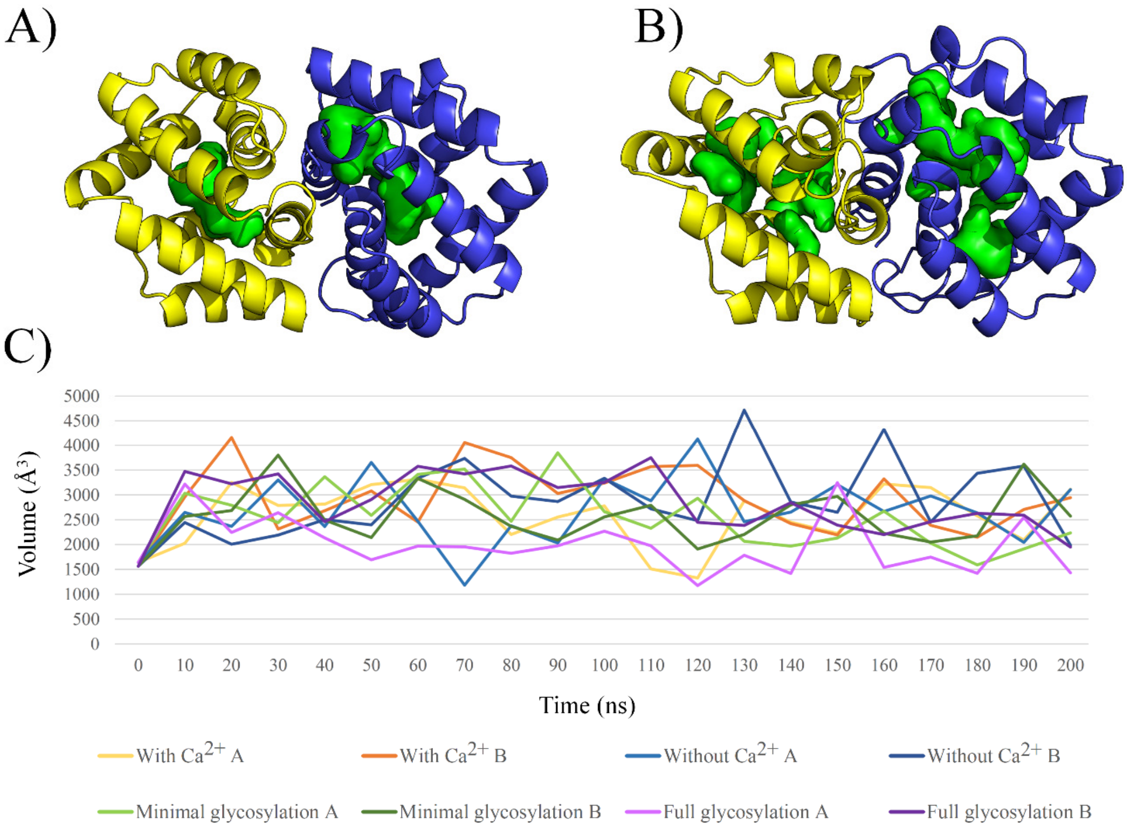

Fig. S6. Cavity volume overview (calcium-free system as an example). (A) initial cavity volume, (B) final cavity volume (cavity volumes shown as green surfaces); (C) cavity volume measurements for each Fel d 1 chain in four simulations.
