# Supplementary material for: The Calcium Goes Meow: Effects of Ions and Glycosylation on Fel d 1, the Major Cat Allergen
Source: PLoS One. 2015 Jul 2;10(7):e0132311. doi: 10.1371/journal.pone.0132311 (PMC4489793; doi:10.1371/journal.pone.0132311)
Supplement: S7 Fig — The distance between each original chain in the native Fel d 1 (half chains of the crystallographic protein) is shown. The oscillation may indicate an opening-closing behavior. (calcium-free system in black, calcium-bound system in red, minimally glycosylated system in blue, fully glycosylated system in green; distances measured for the centers of mass in each structure). (PDF) [file pone.0132311.s007.pdf]

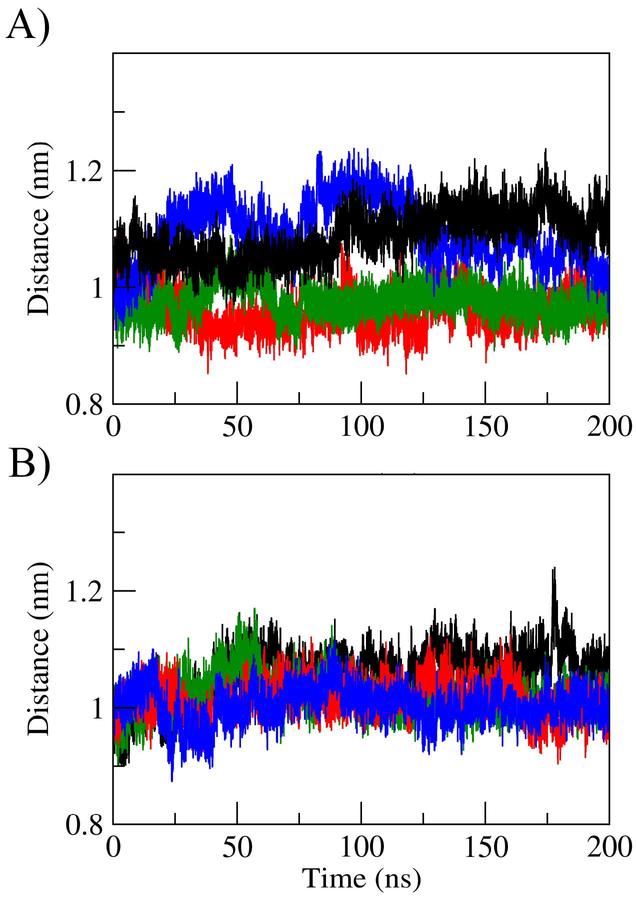


**Fig. S7. Structural 'oscillation' observed for (A) chain A, and (B) chain B of Fel d 1 in four different simulations.** The distance between each original chain in the native Fel d 1 (half chains of the crystallographic protein) is shown. The oscillation may indicate an opening-closing behavior. (calcium-free system in black, calcium-bound system in red, minimally glycosylated system in blue, fully glycosylated system in green; distances measured for the centers of mass in each structure).
