# Supplementary material for: The Calcium Goes Meow: Effects of Ions and Glycosylation on Fel d 1, the Major Cat Allergen
Source: PLoS One. 2015 Jul 2;10(7):e0132311. doi: 10.1371/journal.pone.0132311 (PMC4489793; doi:10.1371/journal.pone.0132311)
Supplement: S1 Table — These encompass atom types, atomic partial charges, charge-group definition, bond stretching, bond-angle bending, improper dihedral deformation and torsional potential dihedral. (PDF) [file pone.0132311.s008.pdf]

Table S1. Parameters used in the GROMOS 53A6glyc force field for sialic acid, in a GROMACS-compatible format. These encompass atom types, atomic partial charges, charge-group definition, bond stretching, bond-angle bending, improper dihedral deformation and torsional potential dihedral.

```
[ NEUN ]
 [ atoms ]
   C5   CH1     0.00000     0
   N5     N    -0.28000     0
  HN5     H     0.28000     0
  C5A     C     0.38000     0
  O5A     O    -0.38000     0
  C5B   CH3     0.00000     0
   C4   CH1     0.23200     1
   O4    OA    -0.64200     1
  HO4     H     0.41000     1
   C7   CH1     0.23200     2
   O7    OA    -0.64200     2
  HO7     H     0.41000     2
   C8   CH1     0.23200     3
   O8    OA    -0.64200     3
  HO8     H     0.41000     3
   C9   CH2     0.23200     4
   O9    OA    -0.64200     4
  HO9     H     0.41000     4
   C6   CH1     0.37600     5
   O6    OA    -0.48000     5
   C2   CH1     0.23200     5
   O2    OA    -0.53800     5
  HO2     H     0.41000     5
   C3   CH2     0.00000     5
   C1     C     0.36000     6
  O1A    OM    -0.68000     6
  O1B    OM    -0.68000     6
[ bonds ]
   C5    N5    gb_21
   N5   HN5    gb_2
   N5   C5A    gb_10
  C5A   O5A    gb_5
  C5A   C5B    gb_16
   C5    C4    gb_26
   C5    C6    gb_26
   C4    O4    gb_20
   C4    C3    gb_26
   O4   HO4    gb_1
   C3    C2    gb_26
   C7    C6    gb_26
   C7    O7    gb_20
   O7   HO7    gb_1
   C7    C8    gb_26
   C8    O8    gb_20
   O8   HO8    gb_1
   C9    C8    gb_26
   C9    O9    gb_20
   O9   HO9    gb_1
   C6    O6    gb_20
   O6    C2    gb_20
   C2    O2    gb_20
   O2   HO2    gb_1
   C1   O1A    gb_6
   C1   O1B    gb_6
   C1    C2    gb_26
[ angles ]
;  ai    aj    ak   gromos type
   C6    C5    N5     ga_15
   N5    C5    C4     ga_15
   C5    N5   HN5     ga_18
   C5    N5   C5A     ga_31
  HN5    N5   C5A     ga_32
   N5   C5A   O5A     ga_33
   N5   C5A   C5B     ga_19
  O5A   C5A   C5B     ga_30
   C4    C5    C6     ga_8
   C5    C4    O4     ga_9
   C5    C4    C3     ga_8
   O4    C4    C3     ga_9
   C4    O4   HO4     ga_12
   C4    C3    C2     ga_8
   C5    C6    C7     ga_8
   C5    C6    O6     ga_9
   C7    C6    O6     ga_9
  HO7    O7    C7     ga_12
   O7    C7    C6     ga_9
   O7    C7    C8     ga_9
   C6    C7    C8     ga_8
  HO8    O8    C8     ga_12
   O8    C8    C7     ga_9
   O8    C8    C9     ga_9
   C7    C8    C9     ga_8
   O9    C9    C8     ga_9
  HO9    O9    C9     ga_12
   C6    O6    C2     ga_10
   C3    C2    O6     ga_9
   C3    C2    O2     ga_9
   O6    C2    O2     ga_9
   O6    C2    C1     ga_9
   O2    C2    C1     ga_9
   C2    O2   HO2     ga_12
  O1A    C1   O1B     ga_38
  O1A    C1    C2     ga_22
  O1B    C1    C2     ga_22
   C3    C2    C1     ga_8
 [ impropers ]
;  ai    aj    ak    al   gromos type
   N5   C5A   C5B   HN5     gi_1
  C5A   C5B    N5   O5A     gi_1
   C5    O6    C7    C6     gi_2
   C5    O4    C3    C4     gi_2
   C5    N5    C4    C6     gi_2
   C2    O6    O2    C3     gi_2
   C3    O6    C1    C2     gi_2
   C1   O1A   O1B    C2     gi_1
[ dihedrals ]
;  ai    aj    ak    al   gromos type
   C3    C4    C5    N5     gd_16
   C7    C6    C5    N5     gd_16
   O4    C4    C5    N5     gd_17
   O6    C6    C5    N5     gd_18
   C4    C5    N5   C5A     gd_39
   C5    N5   C5A   C5B     gd_14
   C6    C5    C4    O4     gd_43
   C6    C5    C4    O4     gd_44
   C6    C5    C4    C3     gd_34
   C4    C5    C6    C7     gd_34
   C4    C5    C6    O6     gd_43
   C4    C5    C6    O6     gd_44
   C3    C4    O4   HO4     gd_30
   C5    C4    C3    C2     gd_34
   O4    C4    C3    C2     gd_43
   O4    C4    C3    C2     gd_44
   C4    C3    C2    O6     gd_43
   C4    C3    C2    O6     gd_44
   C4    C3    C2    O2     gd_43
   C4    C3    C2    O2     gd_44
   C6    C7    O7   HO7     gd_30
   O7    C7    C6    O6     gd_5
   O7    C7    C6    O6     gd_37
   C6    C7    C8    O8     gd_43
   C6    C7    C8    O8     gd_44
   O7    C7    C8    O8     gd_18
   C7    C8    O8   HO8     gd_30
   C7    C8    C9    O9     gd_43
   C7    C8    C9    O9     gd_44
   O8    C8    C9    O9     gd_18
   C8    C9    O9   HO9     gd_30
  O1A    C1    C2    C3     gd_40
   C5    C6    O6    C2     gd_29
   C6    O6    C2    C3     gd_29
   O6    C2    O2   HO2     gd_2
   O6    C2    O2   HO2     gd_32
 [ exclusions ]
  HO2    O6
```
