# Supplementary material for: The Calcium Goes Meow: Effects of Ions and Glycosylation on Fel d 1, the Major Cat Allergen
Source: PLoS One. 2015 Jul 2;10(7):e0132311. doi: 10.1371/journal.pone.0132311 (PMC4489793; doi:10.1371/journal.pone.0132311)
Supplement: S2 Table — Nodes that appear more than once are grouped by color. Interactions detected between pairs by PDBsum for the crystallographic structure are in parentheses. (PDF) [file pone.0132311.s009.pdf]

Table S2. Essential node pairs detected in four Fel d 1 simulated systems. Nodes that appear more than once are grouped by color. Interactions detected between pairs by PDBsum for the crystallographic structure are in parentheses.

	**Interface (Chain A - Chain B)**	**Chain A**	**Chain B**
Without Ca^{2+}	Lys29-Asp82 (1HB, NBC)<br>Leu132-Leu94	Cys44-Cys118 (DS)<br>Ile64-Val102	Leu41-Tyr119 (NBC)<br>Phe85-Met134 (NBC)
With Ca^{2+}	Leu31-Leu94<br>Asn91-Leu129 (NBC)<br>Leu132-Phe85 (NBC)	Cys3-Ala74<br>Val10-Phe84	Val6-Tyr81<br>Asn37-Arg127
Minimal Glycosylation	Phe85-Asp130 (NBC)<br>Val128-Leu94<br>Ser139-Thr76 (NBC)	Val6-Phe80<br>Ile40-Asn122 (NBC)	Val6-Phe80<br>Asn37-Arg127
Full Glycosylation	Phe85-Leu132 (NBC)<br>Asn89-Leu129 (NBC)<br>Thr135-Phe85 (NBC)	Lys7-Phe80<br>Asn37-Arg127 (2HB)	Cys3-Tyr65<br>Asn37-Val128

HB: hydrogen bond(s), NBC: non-bonded contacts, DS: disulfide bridge.
Note: There is a pair in the simulation with Ca^{2+} (Leu129B-Arg127B) that connects the interface pair (Asn91-Leu129) to the 'hinge' pair (Arg127-Asn37).
