# Supplementary material for: The Calcium Goes Meow: Effects of Ions and Glycosylation on Fel d 1, the Major Cat Allergen
Source: PLoS One. 2015 Jul 2;10(7):e0132311. doi: 10.1371/journal.pone.0132311 (PMC4489793; doi:10.1371/journal.pone.0132311)
Supplement: S3 Table — (PDF) [file pone.0132311.s010.pdf]

Table S3. Sizes of simulated systems.

System		Number of atoms	Number of counterions (Na^{+})
AMBER	With Ca^{2+}	28,951	8
	Without Ca^{2+}	31,382	16
CHARMM	With Ca^{2+}	31,750	8
	Without Ca^{2+}	31,382	16
GROMOS	With Ca^{2+}	34,627	8
	Without Ca^{2+}	28,559	16
Crystal mimic	With Ca^{2+}	586,592	88
Glycosylated	Full glycosylation	88,194	14
	Minimal glycosylation	65,078	8
